# Current understanding and management of *Helicobacter pylori* infection: an updated appraisal

**DOI:** 10.12688/f1000research.14149.1

**Published:** 2018-06-11

**Authors:** Shamshul Ansari, Yoshio Yamaoka

**Affiliations:** 1 Department of Environmental and Preventive Medicine, Oita University Faculty of Medicine, 1-1 Idaigaoka, Hasama-machi, Yufu-City, Oita, 879-5593, Japan; 2 Department of Medicine-Gastroenterology, Baylor College of Medicine, 2002 Holcombe Boulevard, Houston, TX, 77030, USA

**Keywords:** Helicobacter pylori, virulence factors, eradication therapy, antibiotics resistance

## Abstract

In addition to its role in gastric conditions, *Helicobacter pylori* has been found to contribute to the development of several non-gastric issues in recent years. Eradication therapy is the only effective management strategy to minimize the *H. pylori*-related gastric cancer and extra-gastric complications. For an effective “test and treat” strategy, diagnosis and therapy are both important. Because the infection is usually asymptomatic, patient selection is a critical issue for timely diagnosis and many clinical and demographic factors should be considered. Clarithromycin and metronidazole resistance rates also need to be considered while eradication therapy is offered. In this report, we discuss the issues which must be taken into account for the correct and timely diagnosis and for the antibiotic therapy-based management of *H. pylori* infection.

## Introduction

*Helicobacter pylori* is the causative agent of chronic gastric infections, and it has been estimated that at least half of the world’s population is infected. A recent meta-analysis on the global prevalence of *H. pylori* infection has shown an overall prevalence of 44.3%, and estimated prevalences are as high as 89.7% in Nigeria and as low as 10.0% in Indonesia and 8.9% in Yemen ^1^. Socio-economic status, together with the level of urbanization and sanitation conditions, likely reflects the differences of *H. pylori* prevalence from country to country ^2^. The exact route of this bacterium’s transmission is unclear; however, evidence supports person-to-person transmission via oral–oral or fecal–oral route between family members ^3, 4^. After it has transited to the gastric lumen, *H. pylori* localizes to specific locations such as the antrum and corpus, where it is well adapted to survive in acidic conditions and establish persistent infection ^5^. Once infection is established, several gastro-duodenal complications such as gastritis, gastric ulcer, duodenal ulcer, dyspeptic symptoms, gastric cancer, and gastric mucosa-associated lymphoid tissue (MALT) B-cell lymphoma may develop ^6^. Gastric cancer persists as a major public health issue and ranks as the third most common cause of cancer-related mortality; in 2012, it led to the deaths of about 723,100 individuals ^7, 8^. In addition to its association with gastro-duodenal complications, *H. pylori* in recent years has been reported to cause several extra-gastric complications.

Epidemiological studies have suggested an association between *H. pylori* infection and certain other extra-gastric complications such as ischemic heart disease, neurodegenerative diseases, and hematological disorders (iron deficiency anemia, immune-thrombocytopenic purpura, and vitamin B _12_ deficiency) ^6, 9, 10^. Bellos *et al*. recently found that *H. pylori* infection in pregnant women increases the risk of developing preeclampsia, which is a potent contributor to maternal and fetal morbidity and mortality ^11^. Another complication, hyperemesis gravidarum, can be found in up to 2.0% of women with early pregnancy and its onset has been associated with *H. pylori* infection ^12^. Cen *et al*., in a meta-analysis comprising 18 studies involving 1,544 participants, found an overall threefold increased risk for gall bladder disease, such as cholecystitis and cholelithiasis, in association with *H. pylori* infection. In Asian populations, the risk is higher than in non-Asian populations ^13^. Serological evidence for *H. pylori* infection was found to be associated with the development of hepatic diseases such as non-alcoholic fatty liver disease ^14^. With regard to the conclusive evidence linking *H. pylori* infection with hematological disorders (iron deficiency anemia, immune-thrombocytopenic purpura, and vitamin B _12_ deficiency), the Maastricht V/Florence consensus recommended *H. pylori* eradication therapy for these complications in addition to the gastric complications ^15^.

Eradication therapy significantly decreases the risk of developing gastric cancer if given before the onset of pre-cancerous lesions (atrophy, intestinal metaplasia, and dysplasia) ^16^ and has proven to be the only effective strategy for reducing the development of gastric cancer. When a population-based “test and treat” strategy in a geographic region is being considered, which tests are preferred for the diagnosis of *H. pylori* infection, which subjects should be offered the diagnosis, and which treatment should be prescribed remain critical issues. The main aim of this review is to summarize the information regarding the strategic approaches and indications for the diagnosis of *H. pylori* as well as appropriate antibiotic therapy-based management.

## Virulence factors implicated in gastro-duodenal diseases

Although a declining trend of *H. pylori* infection has been reported in many countries, the incidence of gastric cancer remains a major public health issue for cancer-related deaths worldwide ^7^. Despite the role of host factors and environmental conditions of the stomach, bacterial virulence factors play an important role in *H. pylori*-related pathogenicity. The virulence factors such as cytotoxin-associated gene A (CagA) and vacuolating cytotoxin A (VacA) are the most studied and closely associated with gastric epithelial cell apoptosis and the development of severe gastric complications ^17, 18^. CagA is an oncogenic protein that possesses an EPIYA (Glu–Pro–Ile–Tyr–Ala) motif; after CagA’s internalization in the host epithelium by the type 4 secretory system (T4SS), which forms a needle-like structure ^19^, the tyrosine of the EPIYA motif undergoes phosphorylation. CagA can possess four different types of EPIYA motifs—EPIYA-A, -B, -C, and -D—depending on the geographic region. *H. pylori* strains from Western countries usually possess CagA with EPIYA-A, -B, and -C (one to three EPIYA-C), whereas those from most of the East Asian countries possess EPIYA-A, -B, and -D. EPIYA-A and -B are carried by almost all CagA, and the third EPIYA motif (C or D) is a geographic, genotypic, and virulence characteristic ^20^. The presence and characteristics of the third EPIYA motif (EPIYA-C or -D) determine the virulent characteristics of CagA. In a recent meta-analysis, CagA with a single EPIYA-D motif was significantly associated with the development of gastric cancer while CagA with multiple EPIYA-C motifs was found to be a significant risk factor for peptic ulcer disease (PUD) in Asian countries; however, in the US and Europe, CagA with multiple EPIYA-C motifs was associated with the development of gastric cancer ^21^. The VacA is an exotoxin which affects multiple cellular pathways and induces host cell vacuolation and cell death (reviewed in 22).

Blood group antigen-binding adhesin (BabA) is a major outer membrane protein and another major virulence factor that is involved in the attachment of bacteria to the host epithelium, which leads to double-strand DNA breaks and translocation of CagA to the host cells ^23, 24^. The specific location of *bab-*paralogous genes in three loci ( *babA/babB/-*) was found to be associated with the development of pre-cancerous lesion (atrophy) and peptic ulcer ^25^. The role and characteristics of many other proteins have been implicated in the development of *H. pylori-*related pathogenicity. The outer inflammatory protein A (OipA), duodenal ulcer-promoting gene A (DupA), sialic acid-binding adhesin (SabA), and protein which is induced by contact with epithelium (IceA) are implicated in the triggering of gastric epithelial cell apoptosis and the development of severe gastric complications such as peptic ulcer and gastric cancer ^26– 30^.

In addition to its acid-neutralization function, urease, a potent virulence factor, was recently reported to induce angiogenesis, the formation of new blood vessels from pre-existing vasculature, which is important for tumor growth and metastatic dissemination and plays a key role in the progression of gastric cancer ^31, 32^. In a study using *in vitro* endothelial cell tube formation assay and *in vivo* chorioallantoic membrane (CAM), the addition of *H. pylori* urease was found to induce the formation of tube-like structures by human umbilical vascular endothelial cells and CAM, respectively ^33^. Another gene, *hp0169*, the only gene annotated as collagenase in *H. pylori* that encodes the protein HpPrtC, which belongs to the protease family, was found to affect pathogenicity through cell viability, proliferation, and apoptosis ^34^. The *H. pylori* strains harboring these virulence factors are considered more pathogenic than the strains lacking these factors. Therefore, evaluation of these virulence factors provides insight for risk stratification and clinical outcome.

## Diagnostic approaches for *H. pylori* infections

Currently, the diagnosis of *H. pylori* infection is carried out by invasive (for example, endoscopy and endoscopic biopsy for histopathology, culture, and rapid urease test) and non-invasive (for example, urea breath tests, stool antigen test, and serological tests) methods ^35^. However, the diagnostic preferences are based on the prevalence of *H. pylori* infection and age-related gastric cancer incidence in each area. For example, the non-invasive methods are preferred mostly in areas where the gastric cancer incidence is low, whereas endoscopy is recommended in those patients who have a high likelihood of developing gastric cancer, such as those over 60 years of age (or even in younger patients in some European countries), and who have a family history of gastric cancer or are in geographic regions with a high incidence of gastric cancer. The guidelines of the Japanese Society for Helicobacter Research put forth its recommendations suggesting that the diagnosis of *H. pylori* infection is performed by using at least one of several invasive and non-invasive methods; however, increased accuracy is obtained by using multiple diagnostic tests ^36^. Despite their high accuracy, the endoscopy-based diagnostic methods are not recommended for screening purposes and this is because of their invasiveness, high cost, and unavailability ^37^. The Maastricht V/Florence consensus report recommended using non-invasive methods such as locally validated serological tests over endoscopic procedures for the diagnosis of *H. pylori* infection in patients with dyspeptic symptoms ^15^. Moreover, the American College of Gastroenterology (ACG) and Canadian Association of Gastroenterology, considering the adverse effects that may occur because of endoscopy, suggested the use of upper gastrointestinal endoscopy in patients who present with dyspeptic symptoms and are over 60 years of age or if the patient belongs to a high-risk family or a region with gastric cancer ^38^. However, in some European countries, endoscopy is recommended in patients over 45 years of age who have predisposing factors such as a high chance of developing gastric cancer ^39^. In this context, the non-invasive methods are considered the preferred and recommended methods for the mass screening of *H. pylori* infection despite the possible drawbacks they may have. For example, the urea breath test is currently recommended as the best approach for the screening of *H. pylori* infection because of its non-invasiveness and high sensitivity ^15^; on the other hand, it is relatively expensive and requires mass spectrometric analysis (which may not be available at resource-limited centers) ^40^, and false-positive and -negative results may occur (albeit rarely). For example, *Neisseria flavescens* and *Pseudomonas fluorescens*, the urease-producing bacteria that were found to colonize the stomach of patients with gastritis, are potential pathogens that can give a false-positive result using the urea breath test ^41, 42^. The stool antigen test is the preferred method for the detection of *H. pylori* infection in children ^43^; however, low sensitivity and specificity have been reported in patients with low bacterial density and in those with peptic ulcer bleeding ^44^. Therefore, the preference of appropriate diagnostic tests depends on many factors such as the patient’s choice and the test’s accuracy and availability as well as its cost-effectiveness.

## Indications for “test and treat” strategy

Almost all *H. pylori*-infected individuals have chronic active gastritis on biopsy, and the clinical outcome of the infection is quite unpredictable, ranging from asymptomatic to a severe complication such as peptic ulcer and gastric cancer; however, these are mostly preventable by eradication therapy ^45^. Several studies have reported that eradication therapy for *H. pylori* in healthy and asymptomatic patients reduces the risk of developing gastric cancer; however, in patients with pre-neoplastic lesions, such as intestinal metaplasia and dysplasia, reversal of this pathological progression was hardly achieved by eradication therapy ^16, 46, 47^. However, reports have found significant improvement in prognosis and reversal of atrophy and even intestinal metaplasia after successful therapy, though to a lesser degree in the case of intestinal metaplasia ^48– 50^. Moreover, a recent clinical trial conducted in South Korea reported that eradication therapy is able to significantly prevent the development of gastric cancer after endoscopic removal of early gastric cancer lesions ^51^. Treatment also reduces the risk of infection transmission from individual to individual, and therefore the financial burden that is associated with *H. pylori* infections may be avoided. The Kyoto global consensus report involving members of the Japanese Society of Gastroenterology, the European Helicobacter Study Group, the Asian Pacific Association of Gastroenterology, the Healthy Stomach Initiative, and the working group members of gastroenterology for International Classification of Diseases-11th revision (ICD-11) recommended screening for *H. pylori* gastritis after the age of 12 years and proposed that all positive cases be treated with eradication therapy even if they have no related symptoms or conditions ^52^. With regard to the Kyoto global consensus report, the Maastricht V/Florence consensus recommended the “test and treat” strategy for patients with dyspeptic symptoms. This report also made an important recommendation that patients with hematological disorders (iron deficiency anemia, immune-thrombocytopenic purpura, and vitamin B _12_ deficiency) be administered eradication therapy because there is considerable evidence linking these complications with *H. pylori* infection ^15^. However, because of the low incidence of *H. pylori*-associated gastric cancer in the US, the ACG recommended testing for *H. pylori* infection in patients with predisposing factors such as PUD, a history of PUD, low-grade gastric MALT lymphoma, or a history of endoscopic resection of early gastric cancer ^53^, whereas the Bangkok consensus report for the Association of Southeast Asian Nations (ASEAN) countries (Indonesia, Thailand, the Philippines, Malaysia, Singapore, Vietnam, Myanmar, Cambodia, Laos, and Brunei) emphasized that *H. pylori* infection is more common in dyspeptic patients than in asymptomatic ones and recommended testing for *H. pylori* infection in patients with chronic dyspeptic symptoms ^54^. Thus, the diagnosis of *H. pylori* infection in a particular geographic region should take into account the prevalence of infection, the incidence of severe complications such as gastric cancer in that geographic region, predisposing factors, and the age of the patient (for example, screening using non-invasive tests in younger patients and endoscopy-based methods in patients in the upper extremity of life, usually over 60 years, or over 45 years in some European countries). Irrespective of the diagnostic methods used, all patients with diagnosed *H. pylori* infection should be offered eradication therapy, which is based on the antibiotic resistance rate of that geographic region.

## Current first-line therapeutic strategies

The therapeutic strategy that is offered as the initial course (first-line) to patients with diagnosed *H. pylori* infection provides the greatest chance for eradication overall. Therefore, the first-line eradication therapy plays a key role in the cure of *H. pylori* infections. Additionally, careful selection of the pertinent first-line therapy is mandatory and this should be based on the local resistance rates of the antibiotic constituents. Clarithromycin (a macrolide) has been an important constituent of *H. pylori* eradication therapy, but proton pump inhibitor (PPI)-clarithromycin-based triple therapy with PPI, clarithromycin, and amoxicillin (or metronidazole where its resistance rate is low) is now recommended as the first-line eradication therapy only when clarithromycin resistance is below 15%. However, if clarithromycin resistance exceeds 15%, bismuth quadruple therapy (bismuth, PPI, tetracycline, and metronidazole) or non-bismuth quadruple therapy (PPI, amoxicillin, clarithromycin, and metronidazole; also known as concomitant therapy) may be offered for 10–14 days as an alternative to first-line triple therapy ^15, 53^. In most of the ASEAN countries, metronidazole resistance is high, and an increasing rate of clarithromycin resistance in recent years confers difficulty in achieving the goal of clarithromycin- and metronidazole-based therapy. A meta-analysis on primary antibiotic resistance conducted in the Asia-Pacific region in 2017 reported an increasing pattern of clarithromycin resistance rate in recent years, whereas metronidazole resistance rates were as high as 75% in Vietnam, 84% in Bangladesh, and 88% in Nepal ^55^. However, in most areas, amoxicillin resistance is rare (below 5%), and in some parts clarithromycin resistance is also lower than 15% ^55^; therefore, PPI-clarithromycin-based triple therapy for 14 days is effective ^54^. Another recent meta-analysis based on randomized controlled trials regarding eradication efficacy found an 84.3% cure rate by sequential therapy with PPI, amoxicillin, clarithromycin, and metronidazole in 2013 and this was superior to 7- or 10-day triple therapy but not to 14-day triple therapy and bismuth- or non-bismuth-based therapy ^56^. The ACG also included sequential therapy—consisting of PPI and amoxicillin for 5–7 days followed by PPI, clarithromycin, and metronidazole for a further 5–7 days—as an option for first-line triple therapy ^53^. The clarithromycin and metronidazole resistance rate in a particular geographic region determines the preferred constituents of eradication therapy. For example, in a geographic region where clarithromycin resistance exceeds 15%, it may be replaced with levofloxacin (a fluoroquinolone), and a levofloxacin-based triple therapy consisting of PPI, levofloxacin, and amoxicillin for 10–14 days or sequential therapy consisting of PPI and amoxicillin for 5–7 days followed by PPI, levofloxacin, and metronidazole for a further 5–7 days may be prescribed as an option for first-line therapy ^53^. However, the efficacy of sequential therapy may vary depending on geographic region and antibiotic resistance rate. In a meta-analysis conducted in China, the authors found that 10-day concomitant therapy was more efficacious than 10-day sequential therapy for infection with metronidazole-resistant strains or together with clarithromycin-resistant strains ^57^. The meta-analysis conducted in the Asia-Pacific region in 2017 also reported that in these countries with clarithromycin resistance higher than 15–20%, clarithromycin-based triple therapy as well as sequential and concomitant therapy showed less than 80% eradication efficacies ^55^. In countries with a high incidence of *H. pylori*-associated gastric cancer and clarithromycin resistance exceeding 15–20%, it is better to use alternative approaches to clarithromycin-based eradication therapy. Finally, after the completion of first-line antibiotic treatment, the eradication therapy’s efficacy should be assessed using the urea breath test ^15^. In agreement with the development of multi-drug resistance in other bacterial species, antibiotic resistance in *H. pylori* is an increasing trend because of the overuse and misuse of antibiotics for the treatment of other infections, especially in developing countries ^58^. Currently, the novel polymerase chain reaction-based approach is sensitive for the detection of *H. pylori* DNA in stool samples together with detecting mutations causing clarithromycin resistance ^59^. This non-invasive method could be able to significantly decrease endoscopy-based biopsy sampling for antibiotic resistance determination.

## Geographic distribution of clarithromycin and metronidazole resistance

**Figure 1.**
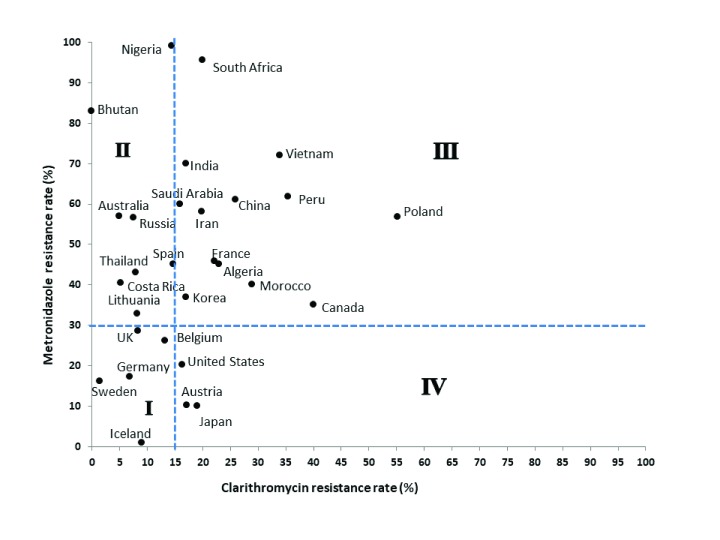
Geographic distribution of clarithromycin and metronidazole resistance. The dotted lines show the threshold levels for clarithromycin and metronidazole resistance rates (15% and 30%, respectively). Both clarithromycin and metronidazole resistance rates are low in countries belonging to area I. Clarithromycin resistance is low but metronidazole resistance is high in countries of area II, whereas in the countries belonging to area III both clarithromycin and metronidazole resistance rates are high. In countries of area IV, the clarithromycin resistance is high but metronidazole resistance is low.

Although the antibiotic resistance rate differs from country to country and even a regional variation may be found within a country, an overall increasing pattern of resistance with time is an emerging problem in many countries ^60^. In 2017, based on the threat that may be imposed, *H. pylori* was listed in the World Health Organization’s “priority list of antibiotic resistance bacteria” and was ranked as top of the most common causes of community-acquired infections if the strain is clarithromycin-resistant ^61^. In general, the clarithromycin and metronidazole resistance rates predict the success rate of standard therapy, as these antibiotics are primary constituents of standard therapy and also resistance to these two antibiotics is frequently seen; therefore, to prescribe the therapy, one must have sound knowledge of regional resistance rates to these antibiotics. In European regions such as Sweden ^62^, Belgium ^63^, Iceland ^64^, Germany ^65^, and the UK ^66^, generally lower resistance rates to both clarithromycin and metronidazole (lower than 15% and 30%, respectively) have been reported ( Figure 1, area I). In countries such as Costa Rica ^67^, Spain ^68^, Nigeria ^69^, and Lithuania ^70^ and in some Asia-Pacific regions such as Thailand, Bhutan, Russia, and Australia ^55^, clarithromycin resistance is lower than 15%; however, metronidazole resistance rates of higher than 30% have been reported ( Figure 1, area II). According to a meta-analysis conducted in Asia-Pacific regions, no clarithromycin resistance was found in Bhutan, although more than 80% of the *H. pylori* strains were metronidazole-resistant ^55^. In Nigeria, metronidazole resistance was reported to be up to 99% ^69^. On the other hand, in South Africa ^71^, Peru ^72^, Algeria ^73^, Canada ^74^, and Morocco ^75^ and in other European countries such as Poland ^76^ and France ^77^, together with other Asia-Pacific regions (for example, India, Iran, Saudi Arabia, South Korea, China, and Vietnam) ^55^, higher resistance rates than the threshold levels for both clarithromycin and metronidazole have been reported ( Figure 1, area III). In most regions, the frequent use of antibiotics is the main contributor to drug resistance and the declining efficacy of eradication therapies. However, hetero-resistance (both resistant and susceptible strains together in one patient’s stomach) has also been reported to contribute to the reduced efficacy of eradication therapy ^65^. The resistance rate of metronidazole usually remains high in developing countries because it is most widely used for the treatment of parasitic infestations, whereas in the developed world its resistance tends to be low. In the US ^78^, Austria ^79^, and Japan ^55^, overall clarithromycin resistance was more than 15%; however, metronidazole resistance was lower than 30% ( Figure 1, area IV).

## Last but not least

Regarding the current therapeutic management of *H. pylori* infections, we, the authors, are deeply concerned with two main points. First, we are well aware that the misuse and overuse of antibiotics pose a great threat to reaching the goal of eradication therapy efficacy and also can create a problem for the future by increasing the rate of antibiotic resistance, as “what does not kill you makes you stronger” and similarly “weaker antibiotics make stronger bacteria”. Thus, the selection of the most appropriate therapeutic strategy based on regional resistance rate is of the utmost importance. Second, *H. pylori* is transmitted from person to person and usually between family members, so there is the possibility of re-infection in cured patients living with other asymptomatic family members (carriers). Therefore, in the authors’ opinion, the “mass eradication” strategy may offer better efficacy of eradication therapy in regions with a high incidence of *H. pylori*-related gastric cancer. In the case of one member being offered eradication therapy owing to some clinical symptoms, the other members (>12 years) of the family should be screened as well and eradication therapy should be offered together to all who are positive for *H. pylori* infection. In this way, the possibility of re-infection from asymptomatic family members is avoided.

## Key points and conclusions

As *H. pylori*-associated gastric complications are a challenging threat to public health, their effective management is of the utmost importance. Diagnosis and therapy are the major arms of management. Non-invasive methods should be the preferred option for diagnosis unless the patient has some predisposing factors necessitating endoscopy. A population-based approach to *H. pylori* eradication should be based on the prevalence of *H. pylori* infection and incidence of gastric cancer in that geographic locality. Moreover, first-line eradication therapy is the most efficacious; therefore, the choice of therapy should be based on the local resistance rate to clarithromycin and metronidazole primarily. Finally, after the completion of therapy, the eradication of *H. pylori* should be assessed.
